# Field quantifications of probability of detection and search patterns to form protocols for the use of detector dogs for eradication assessments

**DOI:** 10.1002/ece3.8987

**Published:** 2022-06-05

**Authors:** Benjamin D. Hoffmann, Craig Faulkner, Laura Brewington, Faye Lawton

**Affiliations:** ^1^ Tropical Ecosystems Research Centre CSIRO Health & Biosecurity Winnellie Northern Territory Australia; ^2^ Reconeco Ecological Consultants Pty Ltd East Lismore New South Wales Australia; ^3^ 10697 East‐West Center Honolulu Hawaii USA; ^4^ Arizona State University Global Futures Lab Tempe Arizona USA; ^5^ Rio Tinto Gove Operations Nhulunbuy Northern Territory Australia

**Keywords:** ants, ecology, Hymenoptera: Formicidae, impacts, invasive

## Abstract

- The use of detector dogs within environmental programs has increased greatly over the past few decades, yet their search methods are not standardized, and variation in dog performance remains not well quantified or understood. There is much science to be done to improve the general utility of detector dogs, especially for invertebrate surveys.
- We report research for detector dog work conducted as part of yellow crazy ant eradication. One dog was first used to quantify the probability of detection (POD) within a strictly controlled trial. We then investigated the search patterns of two dogs when worked through sites using different transect spacings. Specifically, we quantified their presence within set distances of all locations in each assessment area, as well as the time they took to assess each area. In a GIS, we then calculated the relative percentage of the entire search area within six distance categories, and combined this information with the POD values to obtain a site‐level POD.
- The calculated relationship between distance and POD was extremely strong (*R* ^2^ = 0.998), with POD being 86% at 2 m and 28% at 25 m. For site‐level assessments conducted by the two dogs, both dogs achieved the highest site‐level POD when operated on the lowest transect spacing (15 m), with POD decreasing significantly as transect spacing increased. Both dogs had strong linear relationships between area assessed and time, with the area assessed being greater when the transects had greater spacing. The working style of the two dogs also resulted in significantly different assessment outcomes. In 1 h one dog could assess approximately 9.2 ha with transects spaced 20 m apart, and 6.8 ha with transects spaced 15 m apart, whereas the second dog could only assess approximately 6.9 ha with transects spaced 20 m apart, and 4.9 ha with transects spaced 15 m apart.
- Our study provides insight into the ability of dogs to detect yellow crazy ants, and sets the basis for further science and protocol development for ant detection. With the lessons learned from this work, we then detail protocols for using detector dogs for ant eradication assessments.

## Graphical abstract

Our study quantifies the ability of dogs to detect yellow crazy ants, further quantifies site‐level probabilities of detection for eradication determinations, and highlights the basis for further science and protocol development for ant detection.

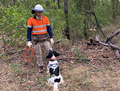

## 1 INTRODUCTION

The use of detector dogs within environmental programs has increased greatly over the past few decades because of their general superiority for detection and cost‐effectiveness compared to humans or other methods (Ballouard et al., 2019; Cristescu et al., 2015; Grimm‐Seyfarth et al., 2021; Orkin et al., 2016), especially when targets are at very low densities (Cheyne, 2011; Russell et al., 2008). But despite the acknowledgment of superior dog abilities to find many target species, background science quantifying the efficacy and limitations of detector dogs to detect the broad array of taxa is lacking, search methods as well as research methods to determine efficacy and limitations are not standardized, and variation in dog performance remains not well quantified or understood (Bennett et al., 2020; Johnen et al., 2017). Clearly, there is much science to be done to improve the utility of detector dogs for conservation.

Detector dogs are now an integral part of ant eradications globally because the areas to be assessed are too large for human searches alone. Within Australia, dogs have been used to declare red imported fire ant *Solenopsis invicta* eradicated from multiple locations for nearly the past two decades and continue to conduct post‐treatment assessments within the national fire ant eradication program in Brisbane (Wylie et al., 2016). These same dogs have also been cross‐trained and used for eradications of browsing ant *Lepisiota frauenfeldi* elsewhere in Australia (Biosecurity Queensland, 2018). Other dogs are used as part of a little fire ant *Wasmannia auropunctata* eradication program (State of Queensland, 2019). In New Zealand, dogs have been used to aid eradication assessments of Argentine ant *Linepithema humile* (Ward et al., 2016). In the United States, dogs are being used to assess the eradication of Argentine ant from locations on the Channel Islands off the coast of California (Boser et al., 2018), and in a recent eradication declaration for yellow crazy ant *Anoplolepis gracilipes* from Johnston Atoll, it was stated that detector dogs sniffed nearly 193 km without finding any ants (Aisha Rickli‐Rahman, personal communication). But despite this broad‐scale use of detector dogs for ant eradications, apart from one study assessing dog detection abilities at only 3 m (Lin et al., 2011), there are no data yet available outside of the gray literature on their efficacy at varying distances, nor are there standard protocols for their use or guidelines of how their assessments are to be used for eradication declaration.

Here, we detail research gained from detector dog work conducted as part of yellow crazy ant eradication assessments. Our primary objective was to quantify one dog's probability of detection (POD) for yellow crazy ants relative to distance from a transect search line. We then use this information to assess how dog search patterns along transects of different spacing affect site‐level POD through real search areas, as well as how these search metrics vary between two detector dogs. Additionally, we present data on how the relationships between search area and time vary for the two detector dogs. Finally, we discuss the implications of all of these findings for eradication declaration protocols.

## 2 MATERIALS AND METHODS

### 2.1 Study area

**FIGURE 1:**
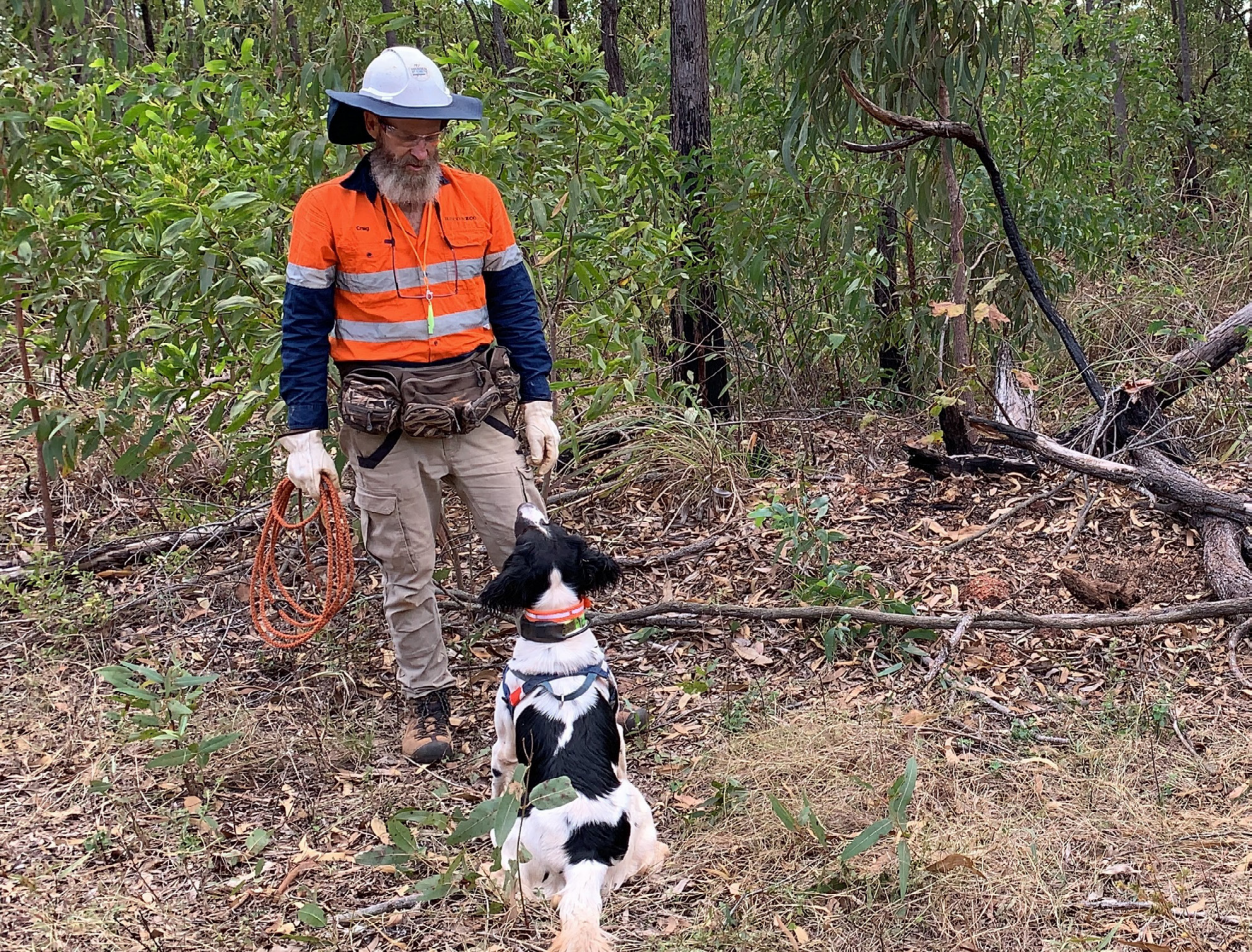
Jet the detector dog with Craig the handler within vegetation with an understorey that was slightly more dense than what was typically utilised for this work

The study was conducted between July and August of 2020 and June and July of 2021, within northeast Arnhem Land near the town of Nhulunbuy (12°11′S, 136°46′E) in Australia's Northern Territory. The regional climate is tropical monsoonal with high temperatures (17–33°C) throughout the year and an annual rainfall of approximately 1200 mm falling predominantly during the summer wet season. The landscape is primarily savanna woodland dominated by *Eucalyptus tetrodonta* (height and canopy cover approximately 15 m and 20%, respectively), with an understory up to 3 m of mainly Acacias and grasses (Williams et al., 1996) (Figure 1). The weather throughout the sample periods was predominantly dry and sunny (working temperature range 18.5–29.5°C), with relatively high humidity (50–92%) and low‐to‐moderate winds (0–6.5 knots). For this work, vegetation with a dense understory was avoided because it was difficult for dogs and the handler to move uninterrupted.

### 2.2 Target species

Yellow crazy ant is one of the world's most invasive ant species (Holway et al., 2002; Wetterer, 2005). It is a medium‐sized (4 mm) species, which in this environment nests at ground level within leaf litter, hollowed wood, or underground holes (Hoffmann, 2015). This species is naturally supercolonial, whereby nests are not discrete entities; instead, workers of all nests display no aggression to each other and will move among nests, thereby forming large, interconnected, and contiguous populations that often cover hundreds of hectares (Haines & Haines, 1978; Hoffmann, 2014; Rao & Veeresh, 1991; Rao et al., 1991). The study region contains many spatially discrete yellow crazy ant populations (Hoffmann & Saul, 2010), many of which have been subject to eradication attempts since 2004 using many baits, treatment timings, and treatment regimes (Hoffmann, 2010), but the success or failure of many of the treatments to achieve eradication remains unknown. It is these treated populations that were used for area‐wide searches to determine if any nests were persisting.

### 2.3 Detector dogs

Jet is a male working‐line Springer Spaniel and is predominantly used for detecting Koala scats in eastern Australia (Figure 1). In 2018, when Jet was 4 years old, he was also trained on the scent of yellow crazy ant. Jet was subsequently used sporadically around Lismore and Terrania in eastern Australia and Nhulunbuy in central north Australia to detect yellow crazy ants. Frankie is a 2‐year‐old female working‐line Springer Spaniel also predominantly used for detecting Koala scats in eastern Australia, and was similarly trained to detect yellow crazy ants in early 2021.

Training the dogs to detect yellow crazy ants was conducted using the following protocols. Unscented cotton pads were first placed in yellow crazy ant nests for periods of between 12 and 36 h to absorb the ant's odor. The pads were then removed using stainless‐steel tongs and placed into double zip‐locked bags. Other pads had ants crushed onto them and were then also placed into double zip‐locked bags. These pads with ant odor were then placed randomly in an open grassy area so that they protruded partially from the ground. Some non‐scented pads were also placed throughout the area. Individually, the dogs were then commanded to search the area and were rewarded (toy reward) when they showed interest in an ant‐scented pad, regardless of whether the odor was sourced from crushed ants or nests. The dogs were not rewarded if they showed interest in an unscented pad. This process was repeated at least twice daily for approximately 4 weeks until the dogs were actively indicating on scented pads only. The second stage of training involved placing 10–50 live ants in small plastic or stainless‐steel containers with a mesh cover. These containers were placed in the field, in both open and more vegetated areas, along with similar containers that did not contain ants. Again, the dogs were commanded to search the area and were only rewarded when they indicated on vessels containing ants. This process was repeated daily for approximately 4 weeks in conjunction with the scent pad training. After the dogs were deemed to be only indicating on yellow crazy ant odor, they were moved to an area where yellow crazy ants were inhabiting and commanded to search the area. When the dogs indicated, whether it be individual ants, numerous ants, or a nest, they were rewarded. This process was repeated at least daily for at least 3 months before they were officially used to detect yellow crazy ants, and then sporadically thereafter in between times they were worked for detecting the ants.

### 2.4 Probability of detection

#### 2.4.1 Experimental design

This work aimed to quantify POD at varying distances under strict time, behavioral, and environmental conditions. Time limitations resulted in this portion of the work only being conducted by one dog, Jet. Locations were selected each day based on the wind direction, with transects being positioned perpendicular to the wind as much as possible. Locations used were roadside edges beside open bushland with very little mid‐story layer (for ease of access); to the best of our knowledge were not populated with *A*. *gracilipes*, and were at least 500 m from known *A*. *gracilipes* populations. It is known that wind variability affects how scent travels through air (Snovak, 2004; Syrotuck, 1972) and such wind variability affects dog detection abilities (Shivik, 2002). Our preliminary work found that wind behavior greatly influenced results, so we created two wind categories: ideal and non‐ideal. Ideal was when the wind at dog level was moving constantly and in a single direction (when overhead winds were around 20 knots). Non‐ideal conditions ranged from calm conditions through to gusty winds up to around 16 knots whereby the wind would also swirl and constantly change direction at dog level. All work was conducted during the cooler parts of the day (from 6:30 to 9:30 a.m. and 3:30 to 6:00 p.m.) when conditions were conducive for yellow crazy ant foraging (Hoffmann, 2015) and also for minimizing panting, which decreases a dog's sniffing rate and scent detection (Gazit & Terkel, 2003).

Stainless‐steel cappuccino shakers (70 mm diameter, 98 mm long) with 50‐mm‐diameter gauzed lids were used as canisters to hold live ants. The ants were collected from a nearby population the day prior, stored overnight in a 20‐L plastic bucket with a moist tissue to prevent dehydration, and placed in the canisters just prior to going to the field assessment locations. Unlike for other utilities like vertebrate scat detection (Mackay et al., 2008), we knew of no prior publications about detection thresholds for dog training or assessments using ants, so we were unsure how many ants would be best to put in the canisters. Preliminary work attempted to use two low ant quantities, 10 and 20 ants, but we observed that Jet continually found the control canisters (no ants), even at great distances, potentially because he was familiar with the canisters from prior work with Koala scats. To help overcome this behavior, we used 50 ants so that the ant scent would be prominent. We also considered that this number could quite likely emulate the odor of hundreds of ants in a nest, which in this region typically has a 20‐mm‐diameter entrance (Hoffmann, 2015).

**FIGURE 2:**
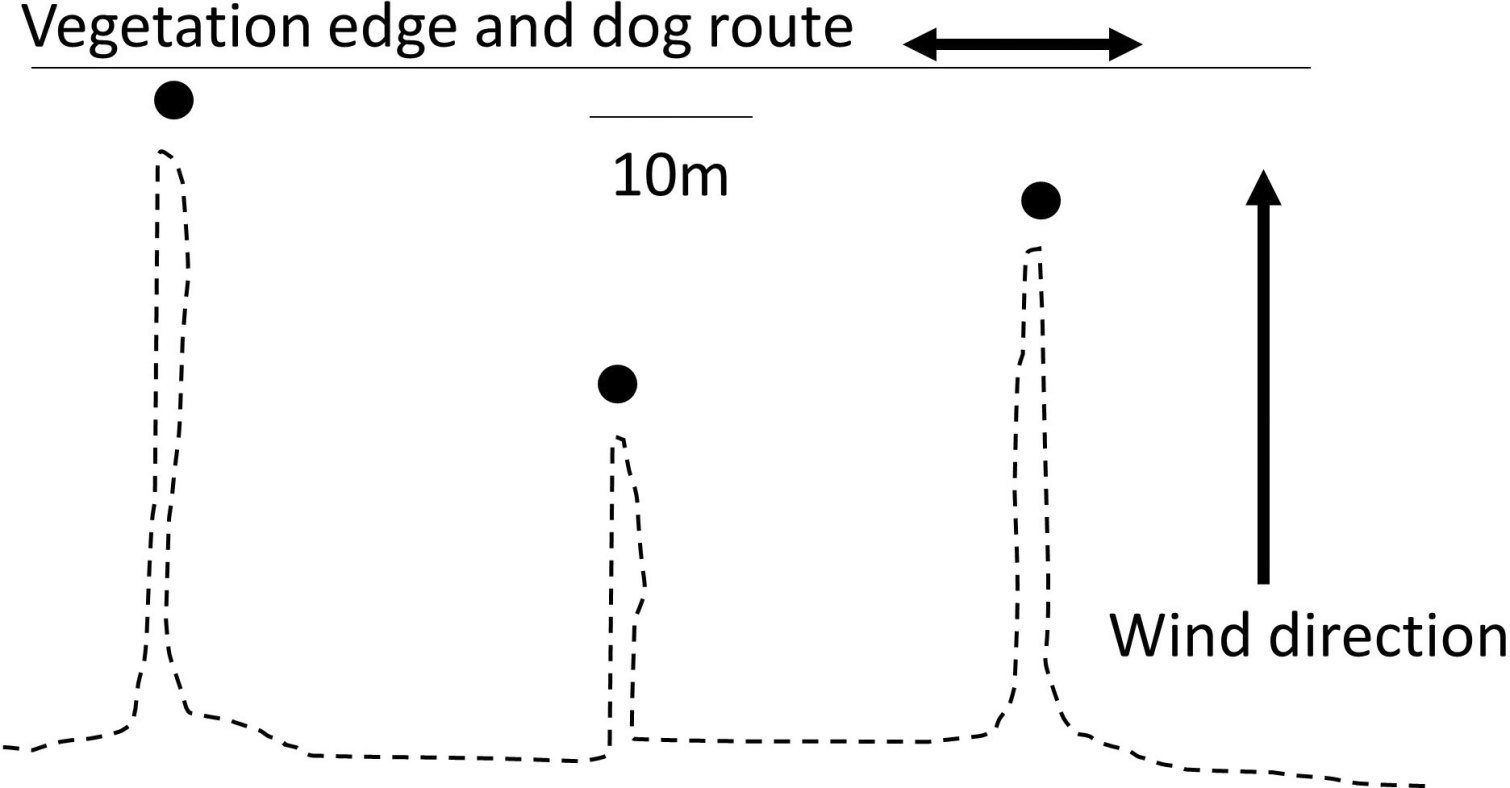
Experimental design. Cannisters (black dots) placed at strategic distances perpendicular to the transect. Dotted lines indicate the walking path of the person setting the cannisters into position in the attempt to prevent the dog from following a human scent to the cannisters

Canisters were spaced at least 30 m apart along the transect, and placed at four distances away from the transect: 2, 10, 20, and 30 m (Figure 2). Preliminary work also found that Jet was very capable of following the path of the person setting the canisters. To prevent this issue, the canisters were set from the opposite side of the assessment area. Separate transects were used for upwind and downwind trials. In all cases, the view of the canister was obscured so that Jet could not simply visually find it. This involved placing it within perennial grass clumps, behind stems, or with a light covering of leaf litter (but not covering the gauze). Both the dog and the handler were not in the area when canisters were laid to prevent bias, nor was the handler told whether canisters were treatments or controls until they were detected by the dog.

Just prior to Jet commencing walking along each transect, environmental conditions were recorded from both the live meteorological bureau data and using a hand‐held Kestrel weather station at approximately 50 cm high (dog height). Data recorded were wind direction and speed, temperature, and humidity.

Jet was tightly controlled to minimize random or false searches and ensure that detections were at the appropriate distance. He was walked on a short leash along a clear path (edge of windrow/vegetation) and was only allowed to walk away from this path greater than the distance of his lead if he pulled strongly. He was walked at a pace of approximately half a meter per second. When given the freedom to pursue a scent, he needed to display an expected zigzag search path, and/or be seen to only be pursuing a canister scent (i.e., not the trail of the person setting the canisters). If Jet was seemingly walking randomly, not following a scent, following the scent of the setter, or pursuing the likes of a prior canister, he was ordered to return to the handler. Also, if he was indeed on the scent of a canister but lost the scent or could not find it, he was promptly called to return.

When Jet found a canister of ants (and signaled by sitting), the trial was paused and he was given a reward, being time to play with his ball. When he found a control canister, the trial did not stop and he was not rewarded, but we noted whether he signaled that he had found yellow crazy ants or not. If he found any canister after being instructed to return to the handler (e.g., he smelled and found a canister farther along the transect rather than one he initially searched for), or if he clearly violated any of the other rules determined above, the found canister was voided from the data. Found canisters were collected immediately. Jet was walked along the entire length of a transect in one direction, and was given a second opportunity in the opposite direction to find any canisters he did not find on the first run.

To additionally prevent Jet from targeting smells from the canisters instead of the ants, individual canisters were only used once per morning or afternoon session, and were later soaked in boiling water for at least 5 min to remove volatiles.

After all voided trials were removed from the data, what we considered minimum sufficient replication (minimum 13 assessments per treatment) was only achieved for non‐ideal conditions because ideal conditions did not occur with sufficient frequency/duration during the assessment period between July 11 and August 25, 2020 (Table 1). Results for the ideal conditions were still analyzed, but must be interpreted with caution. Notably, what we have classed as non‐ideal conditions predominate in this region, so this reflects the on‐ground reality.

**TABLE 1:** Number of replicates achieved for the different treatments: ants present versus controls at the four distances away from the transects in non‐ideal and ideal conditions upwind and downwind of the transects. See also Figure 4 for additional replicates that were voided

Distance (m)	Non‐ideal conditions		Ideal conditions	
Ants present	Upwind	Downwind	Upwind	Downwind
2	14	19	6	3
10	14	16	6	3
20	13	15	7	7
30	15	14	4	7
Controls				
2	14	13	1	1
10	14	13	4	3
20	15	13	5	1
30	14	13	7	4

### 2.5 Coverage and site‐level POD

#### 2.5.1 Experimental design

This work investigated the search patterns of both dogs (Jet and Frankie) by quantifying their presence within set distances of all locations in an assessment area (coverage) when worked through a site using different transect spacings. Multiple areas that had in years prior received toxic treatments to eradicate yellow crazy ant populations (Hoffmann, 2010) were selected for real post‐treatment assessments to determine either persisting presence or absence (eradication) of the ants. Sites were not standardized for size or vegetation, but areas with consistently open ground‐layer vegetation were selected as much as possible to allow free movement of the dogs and the handler. Parallel transects crossing the areas were delineated in the iPhone application iGIS, at distances of 15, 20, or 25 m. Preliminary work found that using an application, whereby the handler could constantly locate themselves relative to the transect line, greatly improved efficiency and accuracy of the work as opposed to just visually estimating the transects. These specific distances were selected to accommodate the dogs’ zigzag search style, which in preliminary observations typically extended about 15 m either side of a transect (influenced by some verbal control from the handler). Note that these transect spacings are the analog of “effective sweep width” used in search theory (Glen & Veltman, 2018). The direction of the transects was not random, but was 45° to the wind to maximize the distance the dog would travel through a scent plume.

The dogs were worked individually for assessments, never together, and always in different areas. Just prior to commencing assessments, the dog that was to conduct the assessment was fitted with a Garmin GPS collar that recorded point locations every 2.5 s. The handler then walked along the transects, using iGIS as a guide, while the dog worked to detect yellow crazy ants. The handler gave no instructions of where to look, only keeping the dog moving along the transect and not too far perpendicularly away from the transect. The handler moved along the transect at the pace of a slow walk, approximately 1 m every 4 s. Assessments were conducted between July 11 to August 25, 2020, and June 23 to July 18, 2021.

Because these were real assessments, there were occasionally instances when persisting ants were detected. In these instances, the work sessions were not used for the coverage assessments because the presence of the ants influenced the dog's search paths.

### 2.6 Area–time relationships

For each of the coverage assessments, the duration of each assessment and distance the dog walked (from the GPS data) were also recorded. The area that the dog assessed was then calculated by multiplying the distance the dog walked by the transect spacing (e.g., 1000 m with 15 m transect spacing = 1.5 ha). Note that this area calculation is what the dog could potentially assess, but does not necessarily reflect the actual area covered (i.e., a dog could walk varying distances within any sized area).

## 3 ANALYSIS

### 3.1 Probability of detection

The percentage of times that canisters containing ants were detected at the four distances, upwind and downwind, and within ideal and non‐ideal conditions, was calculated. Following consideration of the replication of all trials, coupled with the later protocol determination that assessments would be conducted from downwind to upwind within sites, the average of the results for the two upwind situations (ideal and non‐ideal conditions) was used to determine the polynomial of the relationship. This equation was used to calculate POD at exact distances.

### 3.2 Coverage and site‐level POD

At the end of each area assessment session, the GPS tracks of the dog were downloaded, and uploaded into the GIS program ArcGIS 10.6. The paths were then buffered using the following distance categories from the dogs: 0–2 m, 2.01–5 m; 5.01–10 m; 10.01–15 m; 15.01–20 m; and 20.01–25 m. These distance categories were determined subjectively based on a visual assessment of the shape of the POD graph and opinion on how to maximize site‐level POD calculations. From the buffers, we then quantified the relative percentage of the entire search area within each distance category. These relative percentages were then combined with the POD values from the POD graph to calculate a single site‐level POD value for each search, and in some cases where searches were combined, across an entire area of a treated ant population. The exact POD values used for the categories were as follows: 86.0978; 73.796; 56.433; 42.995; 33.482; and 27.894, respectively. So, for example, if all six categories each comprised 20% of the search area, these POD values were each multiplied by 0.2 and summed to give an overall POD of 64.14%. One‐way ANOVAs and Tukey's post hoc tests were used to test for differences in POD between the dog and transect spacing combinations. The assumption of data homogeneity was confirmed using Cochran's tests.

### 3.3 Area–time relationships

The area and time data were plotted separately for both dogs and for transect separations of 15 and 20 m only. The uniformity of the four linear area–time relationships was statistically tested using an analysis of covariance and Tukey's post hoc test. The assumption of data homogeneity was confirmed using a Cochran's test.

## 4 RESULTS

**FIGURE 3:**
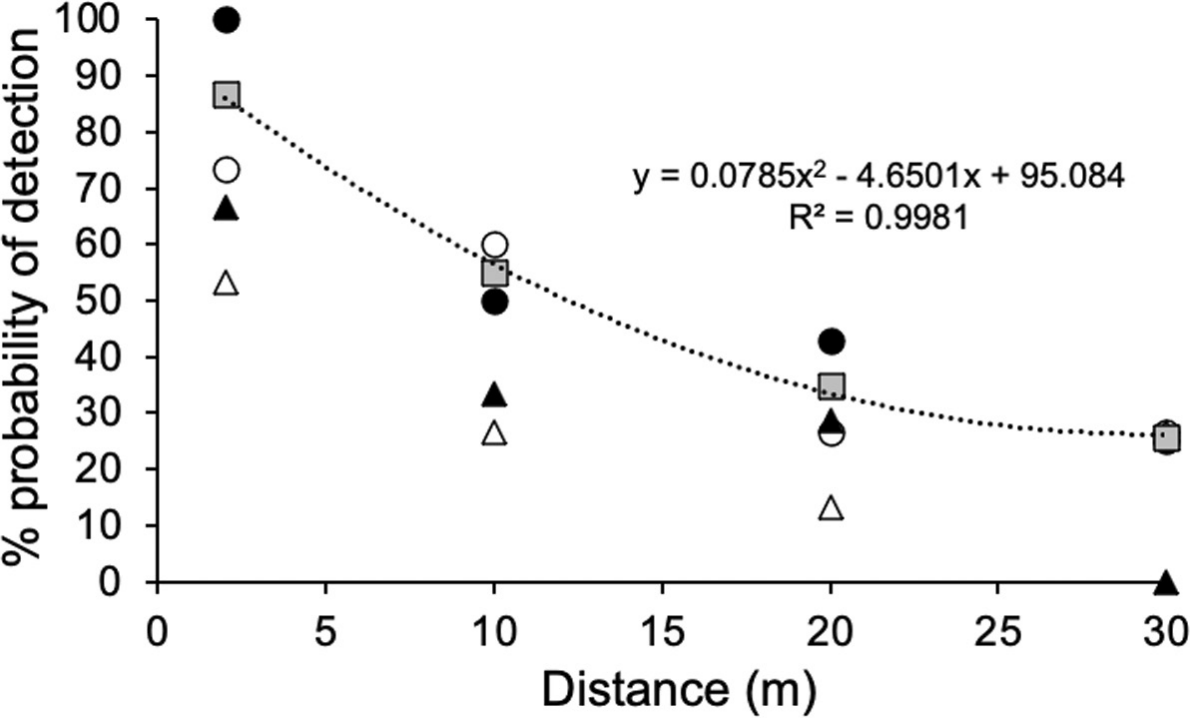
Probability of detection with distance away from the dog within ideal (black) and non‐ideal (white) conditions, with ants upwind (circles) and downwind (triangles) of the dog. Grey squares are the average results for the two upwind outcomes (ideal and non‐ideal conditions) and were used to determine the polynomial of the relationship

**FIGURE 4:**
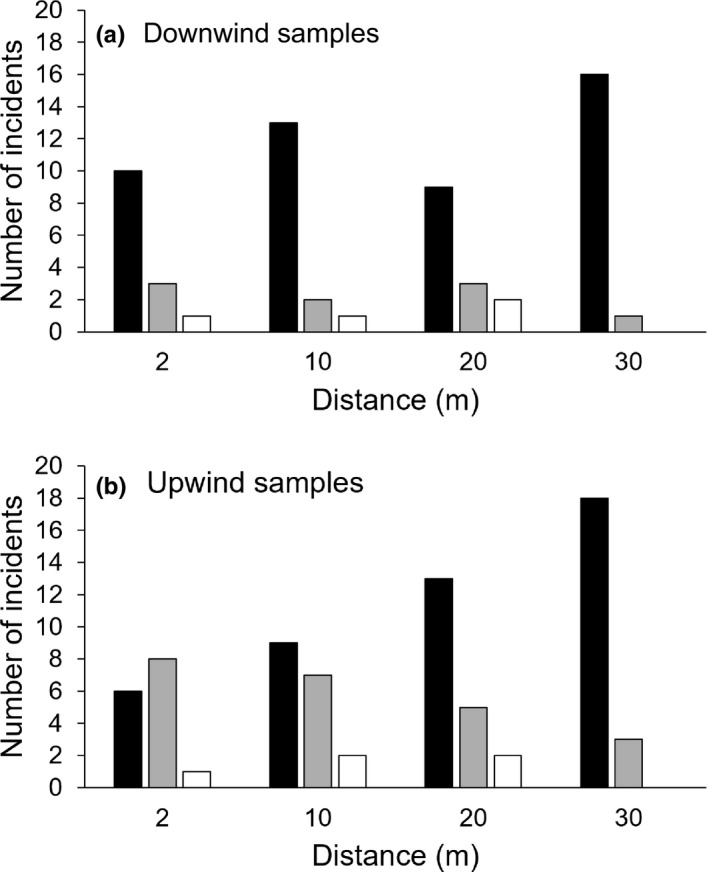
Number of times the dog did not detect a control cannister (black), found a control cannister but did not signal (grey), or found a control cannister and signalled (white) at the four assessment distances when cannisters were placed downwind (a) and upwind (b) of the dog

Jet was clearly able to detect ants both upwind and downwind, including 30 m downwind in non‐ideal conditions (Figure 3). Detectability was greatest at 2 m (86.1%) and declined slightly non‐linearly with distance up to 20 m (33.48%), but was much more plateaued by 30 m (26.23%). Detectability was consistently greater when the ants were upwind of the dog irrespective of the wind conditions. The calculated relationship between distance and detectability was extremely strong (*R* ^2^ = 0.998). Jet did not find the control canisters in 69.6% of the opportunities, but did find and inspect them in 23.7% of opportunities, and found and signaled the presence of ants in 6.7% of opportunities, predominantly (six of nine times) on the first day (Figure 4). Patterns of detection versus non‐detection of control canisters were similar for both upwind and downwind samples, but when the samples were upwind, Jet found and inspected the controls more often, with the finds decreasing with distance.

**FIGURE 5:**
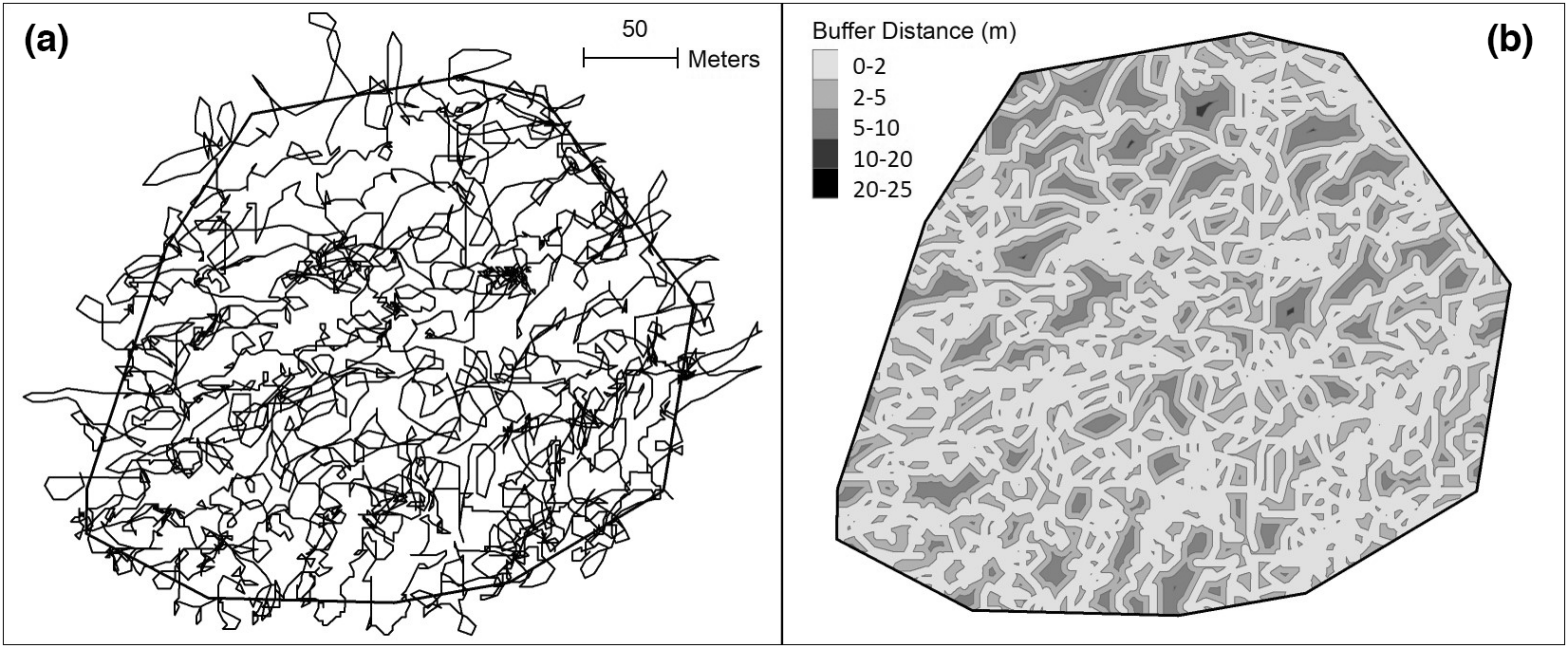
Example of dog tracks (black lines) within a site (black polygon) (a), and the tracks buffered to the five distance categories (b) to quantify the relative percentage of the entire search area within the five distance categories

Both dogs achieved highest site‐level POD when operated using the lowest transect spacing (15 m), with POD decreasing significantly (one‐way ANOVA *F* = 12.4, *p* < .0001) as the transect spacing increased (Table 2). This pattern was probably driven by the percent area within the category of 0–2 m (Figure 5), which also decreased statistically significantly (one‐way ANOVA *F* = 10.16, *p* < .0001) as transect spacing increased (Table 2). For the transect spacings of 15 and 20 m, Frankie achieved 60.9 and 51.5% coverage at <2 m, and Jet achieved 64.5 and 56%, respectively. The reverse pattern typically applied for all other distance categories with percent area within the vicinity of both dogs increasing as transect spacing increased (Table 2).

**TABLE 2:** Experimental design and average (±SE) percentage areas calculated to have been within set distances from the dogs conducting searches along transects of different spacings, as well as average (±SE) calculated probability of detection (POD). Superscript letters indicate statistical separation

Dog	Transect spacing (m)	*n*	% coverage					POD
			0–2 m	2–5 m	5–10 m	10–15 m	15–20 m	
Frankie	15	16	60.9 ± 2.1^acd^	32.8 ± 1.7^abc^	6.3 ± 1.3^ad^	0 ± 0.3^a^	0 ± 0^a^	80.2 ± 0.9^ad^
Frankie	20	5	51.5 ± 2.6^ce^	35.0 ± 0.9^b^	13.2 ± 2.4^be^	0.2 ± 0.6^a^	0.1 ± 0.4^ac^	77.8 ± 1.4^bce^
Jet	15	26	64.5 ± 3^d^	29.2 ± 2.4^c^	6.2 ± 2^a^	0.1 ± 0.4^a^	0 ± 0.2^a^	80.6 ± 1.3^a^
Jet	20	14	56.0 ± 2.6^ae^	33.6 ± 1.7^ab^	10.0 ± 2.1^de^	0.4 ± 0.8^a^	0 ± 0.3^a^	78.8 ± 1.3^cd^
Jet	25	5	45.9 ± 2.5^be^	34.6 ± 1.4^abc^	17.1 ± 2.4^bc^	2.1 ± 1.4^b^	0.2 ± 0.6^bc^	75.7 ± 1.6^be^

**FIGURE 6:**
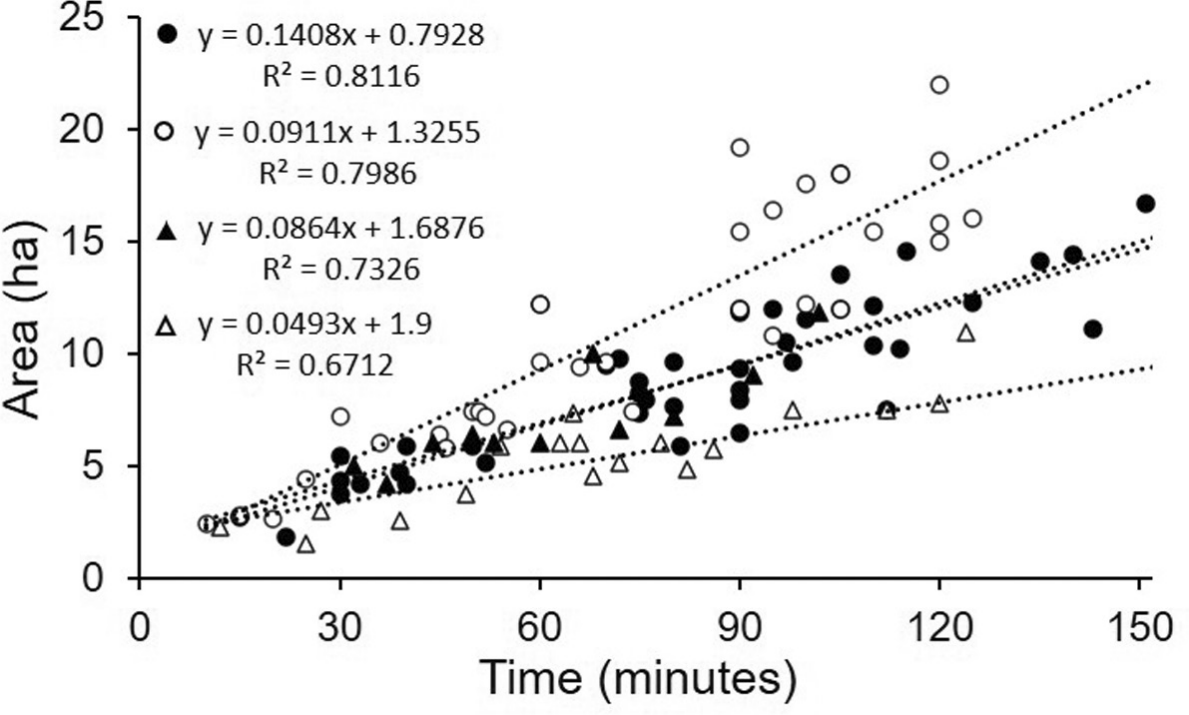
Relationships between time and area for conducting dog assessments with transects spaced at 15 m (white points) and 20 m (black points) for the two dogs: Jet (circles) and Frankie (triangles)

The dogs had combined GPS tracks covering 579.6 km. There were strong linear relationships between area assessed and time, with the area assessed being greater when the transects had greater spacing (Figure 6). Analysis of covariance confirmed that the two transect spacings gave statistically different area/time relationships for the two dogs (*F* = 30.7, *p* < .0001), but that there was no difference between Jet working on 15 m transect spacing and Frankie working on 20 m transect spacing. These different working styles of the two dogs, with Jet moving faster and covering more area than Frankie, meant that in 1 h Jet could assess approximately 9.2 ha with transects spaced 20 m apart, and 6.8 ha with transects spaced 15 m apart, whereas Frankie could only assess approximately 6.9 ha with transects spaced 20 m apart, and 4.9 ha with transects spaced 15 m apart.

## 5 DISCUSSION

### 5.1 Caveats

Our work clearly demonstrated the proficient utility of using detector dogs for ant assessments, and is in accordance with many other studies demonstrating the utility of detector dogs for detecting other flora, fauna, and objects. However, four caveats were associated with this work, some with implications for future work. First, our assessment to determine the baseline POD only utilized one dog, and other dogs would no doubt vary in their POD abilities. To what extent those differences are significant or not, including for between Jet and Frankie, remains unclear. Second, we also only used a single handler, and we recognize that the dog–handler relationship is a fundamental part of assessment outcomes (Jamieson et al., 2018) because the handler is critically important for the likes of seeing, acknowledging, and acting on minor dog signals, assessing whether the dog is potentially underperforming for any reason, as well as affecting dog behaviors. Therefore, the results presented apply only to this handler and these dogs at this time.

Third, it remains unknown just how many ants should be used in POD assessment trials to be relevant to field conditions. Our canisters (70 mm diameter, 98 mm long) with 50 mm diameter gauzed lids) contained only 50 ants, and yellow crazy ant nests would normally contain hundreds to tens of thousands of ants (Haines & Haines, 1978), so we consider that our trials used a much lower ant scent than would probably occur in actual field settings. Indeed, in both years during the real eradication assessments, Jet found ants at such low densities that it was difficult for the handler to confirm their presence, so presumably the dog could detect levels of scent that were much lower than those used in the POD assessments. The only published work we are aware of for ants used between 100 and 10 ants in 50‐ml tubes with a 1.7 × 1.7 mm sieved opening (Lin et al., 2011), but the tubes were used at a set distance of only 3 m, and all tubes containing only 10 ants were found by the dogs. Potentially of relevance is the type of volatiles the dogs are detecting, be it the cuticular hydrocarbons which are generally considered to be the “smell” of the ants (Liang & Silverman, 2000), communication pheromones used extensively by ants (Jackson & Ratnieks, 2006), or a combination of both. Dogs have been able to detect odors at concentrations of only hundreds or thousands of parts per trillion (Kafka, 1997; MacKay et al., 2008) and so it is likely that at small distances dogs will find foraging ants in very low abundance, but dogs will also be able to find ant nests containing hundreds to thousands of workers at much greater distances.

The fourth limitation is that results vary with different environmental conditions and therefore our results were specific to our conditions. The prevailing environmental conditions affect both dog physiological states and scent dispersal. Dogs pant more in higher temperatures (Smith et al., 2003), and because most of the air passes through the mouth rather than the nose when dogs pant (Schmidt‐Nielsen et al., 1970), this results in lower scent detection (Gazit & Terkel, 2003; Gutzwiller, 1990). The same applies for dog fatigue (Homan et al., 2001; Nussear et al., 2008) with fatigued dogs having lower detection rates. For all of our work, we allowed many rests to minimize panting, constantly monitored both dog and handler fatigue, and also restricted work to the relatively cooler parts of the day.

Many environmental conditions affect how scent disperses through the air (Snovak, 2004; Syrotuck, 1972). Multiple studies have found that some variations in wind speed, temperature, and humidity did not influence dog detection efficacy, at least within the environmental condition ranges of these studies. However, it has been found that efficacy is reduced in high humidity (Shivik, 2002), either because dogs get fatigued more quickly or because high humidity saturates the air thereby reducing scent volumes (Pearsall & Verbruggen, 1982). Similarly, it has been found that time to detection increased as wind variability increased (Shivik, 2002), indicating that non‐uniformly directional winds disperse scent non‐linearly, and therefore make it more difficult for a dog to locate the source. Humidity levels in our research area were consistently relatively high, and although not explicitly tested, we did not observe changes in outcomes relative to humidity during the POD assessments. However, we visually observed the effects of different wind conditions, with Jet finding it more difficult to find the ants within highly variable winds, and we quantified this using our two wind categories. Indeed, in the strict POD assessment trial, there were many instances where Jet did detect a scent, but because we restricted him to very limited searching (i.e., he had to demonstrate he was following or homing in on a constant smell, not searching for a smell that he lost), we often stopped his searches, and he therefore found fewer canisters with ants than he could have.

Other environmental factors that can influence detection include topography and vegetation complexity. With limited air movement, scents can pool in depressions (Wasser et al., 2004), and dense vegetation can also cause scents to pool or hang (Snovak, 2004; Syrotuck, 1972). Our study environment had very little topography, the understory vegetation was quite open, and most often, there was air movement sufficient enough, we consider, to prevent scent pooling or hanging, so we are confident that these issues were not present in this study. Regardless, to some extent both dogs and handlers are able to adjust their search behavior if subject to problematic environmental conditions, such as by increasing search time in denser vegetation. Note that we did not alter search behavior among the environmental conditions we assessed, at least not consciously. Ultimately, detection probability appears to be more strongly influenced by distance than by environmental variables, at least at distances less than around 10 m (Reed et al., 2011; Leigh & Dominic 2015).

Fourth, to some extent, Jet may have been detecting the smell of the stainless‐steel canisters in the POD assessment trials. This was especially so on the first day, when he signaled the ant was present at control canisters six of the nine times he incorrectly signaled over the many days the trials were conducted. Given the relatively high number of times that he found the control canisters but did not signal (23.7%), we believe he was indeed smelling many canisters, but rapidly learnt that the ant smell needed to be there for him to signal (and subsequently be rewarded). It is also possible that we were accidentally getting yellow crazy ant scent on the control canisters despite our best attempts to prevent this from occurring. Ultimately, as previously described, Jet had no issues finding yellow crazy ants in such low abundance that it was difficult to confirm the detection, suggesting that any issues with the canisters overall probably did not affect the POD assessments.

### 5.2 POD assessments

The efficacy trial was a very strict linear and time‐controlled trial. It did not represent how a dog would detect ants under normal conditions, in which it is given free rein to find and follow scents, typically zigzagging along and across the path by about 10 m either side, with as much time as is needed to pinpoint a detection. Therefore, we believe our efficacy data can be considered to be absolute minimum values. Similarly, for samples upwind of the dog in ideal conditions, the POD outcome at 10 m of 50% was clearly lower than would be expected most likely due to insufficient replication. Logically, ants upwind should have a higher POD than ants downwind, and we believe this particular POD should be about 75–80%. If so, this would lift the relationship (polynomial equation), and place a 50% POD at about 15 m. This aligns well with the detection of other biota by detector dogs averaging around 10 m (Glen & Veltman, 2018).

Notably, the distances we used were not placed far enough away from Jet to determine when detection approaches zero, so it remains unclear what the distance limitations are. We found that at 30 m Jet still had a 30% POD. For comparison, Cablk and Heaton (2006) found that dogs could detect desert tortoises *Gopherus agassizii* as far as 62.8 m, but Gsell et al. (2010) reported that rodents could be detected much farther, with New Zealand dogs detecting rodents from an average distance of around 50–60 m and up to 150 m. On the other hand, de Oliveira et al. (2012) found that deer (*Mazama* spp.) scats were not detectable farther than 7.2 m. For ants, an improved understanding of these distance limitations will also require an understanding of ant abundance or nest size (volatile volumes). Regardless, knowledge of maximum distance for detection probably has little utility for eradication assessments, for which the sole goal is to maximize POD, even attaining levels of >95%, which clearly requires the dog to be within relatively close proximity to all locations within an assessment area and requiring multiple assessments.

### 5.3 Determination of assessment protocols

Based on the available literature and this study's findings, coupled with knowledge of yellow crazy ant foraging behavior (Hoffmann, 2015), the following protocols were developed for the dog assessments: (1) All work should be conducted at times that are conducive to unabated ant foraging and for minimal dog panting (because dogs can sense smells better when they are not panting). These times around Nhulunbuy are early morning and late afternoons between approximately 6:30–9:30 a.m. and 3:30–6:00 p.m.; (2) Dog paths should be aligned approximately 45° to prevailing winds to maximize the path length within the scent plume and minimize the differences in detectability upwind versus downwind; (3) Transects should be worked from the downwind side of a site to the upwind side so that the dog works toward potential targets; (4) Maximum distance between transects should be 25 m for large sites (around 50 ha and above) and either 20 m or 15 m for smaller sites. These spacings are to be determined by the handler, considering the time available, area to be worked, wind conditions, access conditions, etc., with the focus being to minimize area not accessed within 10 m of the dog; (5) GPS data of dog tracks should be collected as frequently as possible (currently 2.5 s); and (6) Any dog detections must be confirmed by a physical sighting of a yellow crazy ant by the dog handler.

### 5.4 Calculating site‐level POD

For defining protocols to calculate site‐level POD as well as declaring an ant population eradicated, some other factors not yet discussed should also be considered. First, at least for supercolonial ants like yellow crazy ant, it would be very rare that there would be single or disparate ant colonies persisting in a site, especially numerous years after treatments. All data to date from this eradication program clearly show that yellow crazy ant rapidly recovers its populations should eradication fail, with persisting populations covering broad areas and expanding exponentially with time (Hoffmann, 2015), not just existing in point locations. These persisting ants would be very easy for a dog to detect, especially 1–2 years post‐treatment.

Second is a consideration of how many times a dog has an opportunity to assess any point location. The efficacy trial demonstrated what a dog is capable of for a single detection opportunity under strict linear, behavioral, environmental, and time conditions. It is clear from a visual assessment of path maps that any ants at a point location have the potential to be detected on multiple dog paths at multiple distances, both upwind and downwind. Indeed, this opportunity would exist for any point location at least four times, and probably many more times in some instances. Because the POD is cumulative with each independent opportunity, this means that even relatively low probabilities of detection for individual opportunities can result in a high cumulative POD. For example, four opportunities at only 60% probability (8 m in Figure 3) result in a combined 97.44% POD. This is calculated using the equation 1 − (1−*p*)*^n^*, whereby cumulative POD is 1 minus the multiplied probability of failures of detection (i.e., detection failure is 1–*p*, which for 60% POD calculates as 1–0.6 = 0.4, so 1–(0.4 × 0.4 × 0.4 × 0.4) = 0.9744). Four opportunities at only 50% probability (12 m in Figure 3) give a combined probability of 93.75%. Notably, the need for multiple high‐probability opportunities is greatly reduced with just the presence of a single high‐probability opportunity (<10 m). Four opportunities at 10 m with the likely probability of around 70% give a combined POD of 99.19%. On these grounds, it is clear that a dog does not need to conduct multiple assessments over an area to achieve a very high POD, and for eradication to be declared, if the search paths are sufficiently close enough to provide multiple high‐probability opportunities.

### 5.5 Declaring eradication

If the following conditions are met, we believe there is sufficient confidence for declaring the ant population eradicated within an area: (1) If all the assessment protocols are met, and no yellow crazy ants are found; (2) The assessments are conducted at least 1‐year post‐treatment; and (3) A single assessment of a site gives a site‐level POD of around 80% (see Table 2). As argued above, a single site‐level POD calculation only accounts for the closest location a dog was present to any area and does not account for the multiple “independent” opportunities a dog would have to detect a nest as it searches along the multiple transects, which could provide a cumulative site‐level POD of over 95%. However, such a decision is ultimately up to land managers who need to consider the relative costs of conducting more assessments that may be unnecessary versus the cost of re‐treating and re‐assessing if assessments prove to be inadequate (Rout et al., 2014; Spring & Chaco, 2015).

### 5.6 Dogs versus humans

Overwhelmingly, dogs have been found to be superior to humans in terms of detection accuracy, area covered per time, and cost effectiveness (Ballouard et al., 2019; Cristescu et al., 2015; Homan et al., 2001; Nussear et al., 2008; Orkin et al., 2016; Savidge et al., 2011; Smith et al., 2001). Although we did not have direct comparative data to compare here for ant assessment work, the senior author's unpublished data of using teams of people in this same region to conduct ant eradication assessments over the past 15 years indicate that per unit time a dog can assess approximately five times the area of a person conducting visual assessments, and most likely has a higher POD. Moreover, human assessments for declaring ant eradication usually involve far more intensive techniques such as the use of lures spaced only a few meters apart throughout the area, which can take a team of five people an entire day to assess just 1 ha (Hoffmann unpublished data).

### 5.7 Future work

There is absolutely no doubt that dogs are highly effective at detecting ants by odor. Our study has provided valuable insight into the ability of dogs to detect yellow crazy ants, and sets the basis for further science and protocol development. Clearly from the literature there are many factors that can influence dog detection abilities, and it will take many more detailed studies to gain a thorough understanding of critical variations in dog abilities, the influence of environmental factors, and the limitations for conducting this work. With that understanding, dog assessments can be modified to improve efficacy, cost‐effectiveness, and overall calculations of POD. But even now without full quantitative assessments, there are clear adaptive management actions that can be applied to searches to address site‐specific and species‐specific factors.

Aside from the usual need for more assessments of both dogs and handlers in a greater variety of environmental conditions, for formal assessments to be conducted on a greater number of species, as well as for more standards to be developed for investigating and reporting detector dog abilities and limitations (Bennett et al., 2020), we think greatest advances for dog detection work would be achieved by research quantifying odor thresholds (e.g., how few ants to place in a canister) and how that affects POD calculations for field assessments in simple environments. Even though POD will clearly be affected by environmental conditions (e.g., wind speed, temperature, vegetation density, and topography), the influence of these factors is highly predictable.

## AUTHOR CONTRIBUTIONS

**Benjamin D. Hoffmann:** Conceptualization (equal); Data curation (equal); Formal analysis (equal); Investigation (equal); Methodology (equal). **Craig Faulkner:** Investigation (equal). **Laura Brewington:** Formal analysis (equal); Writing – original draft (equal); Writing – review & editing (equal). **Faye Lawton:** Conceptualization (equal); Funding acquisition (equal); Project administration (equal); Writing  – review & editing (equal).

## CONFLICT OF INTERESTS

None declared.

## Data Availability

Data are available in Dryad at: https://doi.org/10.5061/dryad.t76hdr83c.
